# Electroacupuncture ameliorates AOM/DSS-induced mice colorectal cancer by inhibiting inflammation and promoting autophagy via the SIRT1/miR-215/Atg14 axis

**DOI:** 10.18632/aging.205236

**Published:** 2023-11-22

**Authors:** Jinxiao Li, Ying Han, Minfeng Zhou, Na Liu, Huarong Li, Guichen Huang, Zhaomin Yu, Dan Luo, Haiming Zhang, Xiangyi Zheng, Fengxia Liang, Rui Chen

**Affiliations:** 1 Department of Integrated Traditional Chinese and Western Medicine, Union Hospital, Tongji Medical College, Huazhong University of Science and Technology, Wuhan, Hubei 430022, China; 2 Hong Kong Baptist University, Hong Kong, China; 3 Rehabilitation Department of Traditional Chinese Medicine, Union Red Cross Hospital, Wuhan 430015, China; 4 Department of Oncology, Hubei Provincial Hospital of Integrated Chinese and Western Medicine, Wuhan 430071, China; 5 College of Acupuncture and Moxibustion and Orthopaedics, Hubei University of Chinese Medicine, Wuhan 430060, China

**Keywords:** EA, SIRT1, colorectal cancer, autophagy, miR-215, inflammation

## Abstract

Colorectal cancer (CRC) is one of the most common tumors of the digestive tract, with the third-highest incidence and the second-highest mortality rate among all malignant tumors worldwide. However, treatment options for CRC remain limited. As a complementary therapy, acupuncture or electro-acupuncture (EA) has been widely applied in the treatment of various inflammation-related diseases, such as obesity, ulcerative colitis and tumors. Although numerous pre-clinical and clinical studies have investigated the beneficial effects of acupuncture on CRC, the mechanism underlying the therapeutic action of EA is largely unknown. Evidence from previous studies has revealed that SIRT1 participates in CRC progression by activating autophagy-related miRNAs. Using azoxymethane/dextran sulfate sodium- (AOM/DSS-) induced colorectal cancer model in mice, we explored whether EA treatment can inhibit inflammation and promote autophagy via the SIRT1/miR-215/Atg14 axis. Our results showed that EA notably alleviated the CRC in mice, by decreasing the tumor number and DAI scores, inflammation, and increasing body weight of mice. Besides, EA increased the expression of SIRT1 and autophagy. Further experiments showed that SIRT1 overexpression downregulated miR-215, and promoted the expression of Atg14, whereas SIRT1 knockdown induced opposite results. In conclusion, EA can ameliorate AOM/DSS-induced CRC through regulating the SIRT1-mediated miR-215/Atg14 axis by suppressing inflammation and promoting autophagy in mice. These findings reveal a potential molecular mechanism underlying the anti-CRC effect of EA indicating that EA is a promising therapeutic candidate for CRC.

## INTRODUCTION

Colorectal cancer (CRC) is among the most prevalent malignant tumor of the digestive system. According to the survey of the World Health Organization, CRC ranks third among the leading cancers worldwide, and the second-leading cause of mortality [1]. In China, CRC was ranked third in terms of prevalence and fifth among the leading causes of cancer-related mortality in 2019 [2]. CRC negatively affects the physical and mental health status of patients. It also imposes a heavy economic burden on the society and family. Inflammation is one of the main causes of tumorigenesis [3], hence can contribute to development of CRC [4]. It has been shown that autophagy can reduce inflammation and enhance the processing and presentation of tumor antigens, thereby promoting the development of anti-tumor immunosuppressive tumors [5].

The *SIRT1*, which is a NAD+ dependent deacetylation enzyme, is an important regulator of cell cycle regulation [6], ageing-associated metabolism [7], inflammation [8], DNA damage and repair [9, 10]. Recent studies have shown that histone deacetylases are potential therapeutic targets in cancer [9, 11, 12]. For instance, *SIRT1* can deacetylate inflammation-linked transcription factors, e.g., NF-κB, to suppress inflammation [13]. Currently, several studies have shown that SIRT1 can regulate the expression of inflammation-related factors to inhibit CRC [14]. The role of SIRT1 in cancer varies depends on its location and cell type [7]. MicroRNAs (miRNAs) are critical regulators of cellular homeostasis and gene expression. They can also modulate oncogenes and tumor suppressor signaling pathways [15, 16], proliferation [17], invasion [18], growth and metastasis [19] of cancer cells. Besides, numerous studies have documented that miRNA are potential biomarkers and therapeutic targets in cancers [20, 21].

Acupuncture, a traditional Chinese medicine-based therapy, has been shown to have fewer side effects and is affordable [22]. It is widely used in contemporary clinical practice [23, 24]. In cancers, acupuncture has many beneficial effects including alleviating adverse effects such as vomiting [25–27], nausea, and anorexia [28] induced by chemotherapy and radiotherapy. It has been shown acupuncture can regulate immune systems and attenuate tumor-associated inflammatory effects and is able to inhibit tumor growth [29]. However, efficacy of acupuncture/EA in animal models of CRC, and the underlying mechanisms have not been fully resolved. In this study, we found that EA improved CRC symptoms, activated SIRT1 and autophagy to inhibit miR-215, leading to the inhibiting of tumor growth in the CRC. This is the first study to demonstrate that EA ameliorates inflammation and promotes autophagy in CRC via SIRT1/miR-215/Atg14 Axis.

## MATERIALS AND METHODS

### Animal models

A total of 100 specific pathogen-free (*SPF*) six-week-old male C57BL/6J mice were bought from SPF (Beijing) Biotechnology Co., Ltd., (Beijing, China; SCXK (Jing) 2019–0010). They were housed under aseptic conditions for one week. All study mice were housed (five per cage) in an air-conditioned room (20 ± 2°C, 12 h light and 12 h dark cycle), allowed free access to food and water ad libitum, and bred in the Experimental Animal Central of Tongji Medical College, Huazhong University of Science and Technology. All experimental protocols and animal handling procedures were approved by the Animal Care and Use Committee (IACUC) of Tongji Medical College, Huazhong University of Science and Technology (IACUC Number: 2756), and were performed in line with the National Institutes of Health Guide for the Care and Use of Laboratory Animals.

### Animals and experimental design

The mice were stratified randomly into the following five groups (20 mice per group): control group, model group, EA group (EA group), EA + SIRT1 inhibitor group (EA + inhibition group), and SIRT1 agonist resveratrol group (resveratrol group). With the exception of the normal group mice, mice in other groups were given a single injection of AOM (10 mg/kg in PBS; i.p.; A5486, Sigma-Aldrich, St. Louis, MO, USA). After one week of rest, all the mice were administered with 2.5% DSS (02160110, MP Biomedicals, Santa Ana, CA, USA) added to their drinking water for one week, followed by DSS-free water for two weeks. The DSS treatment cycle was repeated three times, and the mice were subjected to the interventions simultaneously.

The EA treatment was applied on Zusanli (ST 36) and Fenglong (ST 40) acupoint with sterile acupuncture needles (0.16 × 7 mm, Suzhou Medical Appliance Factory™, Jiangsu, China), which were inserted 3–5 mm into the acupoint. Then, low-frequency EA (2Hz, 1 mA, continuous wave, Shanghai Medical Electronic Apparatus, Shanghai, China) was applied to initiate the treatment. The EA treatment was performed for 20 minutes each time, three times a week for 11 weeks. The SIRT1 inhibitor EX527 (Selleck, 1.25 mg/Kg, S1541, Selleck Chemicals, Houston, TX, USA) [30] was injected intraperitoneally, after which the EA intervention was performed. The SIRT1 agonist resveratrol (Biosharp, 200 mg/Kg, R006891, Rhawn Co., Ltd., Shanghai, China) [31] was administered by gavage.

### Histology and immunofluorescence

The mice were sacrificed, the whole intestine was immediately removed and washed with ice-cold phosphate buffer saline (PBS) and then opened longitudinally. The number of visible tumors on the colorectal surface was counted with naked eyes before the colorectal tissues were made serial sections and stained with H&E.

Immunofluorescence was performed on paraffin-embedded colonic tissue sections. The sections were deparaffinized, rehydrated, and washed in 1% PBS-Tween. After that, they were treated with 3% hydrogen peroxide, blocked with 10% goat serum, and incubated with Beclin1 (A7353, ABclonal, Wuhan, China), P62 (A19700, Abclonal, Wuhan, China) and LC3 (A19665, Abclonal, Wuhan, China) primary antibody in PBS-Tween containing 1% BSA (1:50) at 4°C overnight. Slides were washed and incubated for 1 h with species-specific fluorescently labeled secondary antibodies. The slides were stained with DAPI (ab104139, Abcam, Waltham, MA, USA). Microscopy detection and collect images by fluorescent microscopy.

### Disease activity index (DAI) score evaluation

DAI is used to track the severity of the disease by scoring the extent of body weight loss, hematochezia, and stool trait. The DAI was calculated by grading the following parameters on a scale of 0–4: weight loss (0, ≤1% 1, 1–5% 2, 5–10% 3, 10–15% 4, >15%), hematochezia (0, negative, 1, thimbleful, blood steak; 2, modicum, blood clot; 3, visible bloody stool; 4, gross bleeding) and stool trait (0, normal; 1, soft but still formed; 2, soft and unformed; 3, loose stools; 4, diarrhea). The calculation method was DAI = (weight loss score + hematochezia score + stool trait score)/3.

### Culture and transfection of CRC cells

The human CRC HCT116 and SW480 cells were purchased from the Procell (Procell Life Science and Technology Co., Ltd., Wuhan, China). HCT116 and SW480 cells were inoculated in DMEM (11965092, Gibco, Waltham, MA, USA) enriched with 10% FBS (04-001-1A, BioInd, Kibbutz, Israel) and 1% penicillin/streptomycin (11548876, Gibco, Waltham, MA, USA) under 37°C and 5% CO_2_ conditions. The miR-215 mimics, inhibitor, negative control and SIRT1 plasmids were bought from the TSINGKE (Tsingke, Beijing, China). The transfection experiments were carried out using Lipofectamine 3000 (2067450, Invitrogen, Carlsbad, CA, USA), following the manufacturer’s instructions.

### Small RNA sequencing

DNA extraction, PCR amplification, MiRNA library preparation and sequencing were conducted by Majorbio Bio-Pharm Technology Co., Ltd., (Shanghai, China). Briefly, total RNAs were extracted from 100 mg colorectal tissues. Adaptors were added to both 3′ and 5′ ends, and then subjected to reverse transcription and polymerase chain reaction (PCR) assay. The PCR products derived from the 18- to 30- nucleotide RNA molecules were purified by electrophoresis and sequenced using the Illumina HiSeq 4000 platform. TargetScan and RNAhybrid were used to predict target genes of miRNAs.

### Enzyme-linked immunosorbent assay (ELISA)

After cutting longitudinally and washing out the fecal materials, the colorectal length was measured, chopped into 1–2 cm pieces and homogenized with a tissue homogenizer in PBS or in a tissue lysate solution, centrifuged at approximately 5000 × g for 5 min, assayed immediately or aliquot and stored homogenates at −80°C. The colorectal tissues levels of IL-6 (EMC004, Neobioscience Technology Co., Ltd., Shenzhen, China), IL-17 (EMC008, Neobioscience Technology Co., Ltd., Shenzhen, China), IL-10 (EMC005, Neobioscience Technology Co., Ltd., Shenzhen, China) and TNF-α (EMC102a, Neobioscience Technology Co., Ltd., Shenzhen, China) were determined with ELISA kits according to the manufacturer’s instructions. Experiment was repeated for three times.

### Real-time quantitative PCR

The RNAiso Plus Kit (9109, TaKaRa, Shiga, Japan) was employed to isolate total RNA from the tissues or cell lines following the manufacturer’s guidelines. The isolated RNAs was quantified using the NanoDrop ONE instrument (Thermo Scientific, Waltham, MA, USA). Afterwards, the HiScript cDNA synthesis kit (R323-01, Vazyme Biotech, Nanjing, China) was employed to generate cDNA with the microRNA-specific stem-loop RT primer or relevant primer. Subsequently, the Universal SYBR qPCR Master Mix (Q111-02, Vazyme Biotech, Nanjing, China) was employed to carry out qPCR. The primers (shown in Table 1) of SIRT1, Beclin1, LC3, P62, Atg14, and β-actin were synthesized using the TSINGKE (Tsingke, Beijing, China). The primers for U6 and miR-215 were synthesized and purified by RiboBio company (RiboBio, Guangzhou, China). The relative mRNA expression of target genes was calculated using 2^−ΔΔCT^ method.

**Table 1:** Primer sequences used for quantitative real-time PCR.

**Gene**	**Forward primer**	**Reverse primer**
Mouse-SIRT1	TGCCATCATGAAGCCAGAGA	CATCGCAGTCTCCAAGAAGC
Human-SIRT1	TAGACACGCTGGAACAGGTTGC	CTCCTCGTACAGCTTCACAGTC
Mouse-Beclin1	CAGCCTCTGAAACTGGACACGA	CTCTCCTGAGTTAGCCTCTTCC
Human-Beclin1	CTGGACACTCAGCTCAACGTCA	CTCTAGTGCCAGCTCCTTTAGC
Mouse-LC3	GTCCTGGACAAGACCAAGTTCC	CCATTCACCAGGAGGAAGAAGG
Human-LC3	GAGAAGCAGCTTCCTGTTCTGG	GTGTCCGTTCACCAACAGGAAG
Mouse-P62	GCTCTTCGGAAGTCAGCAAACC	GCAGTTTCCCGACTCCATCTGT
Human-P62	TGTGTAGCGTCTGCGAGGGAAA	AGTGTCCGTGTTTCACCTTCCG
Mouse-β-actin	CATTGCTGACAGGATGCAGAAGG	TGCTGGAAGGTGGACAGTGAGG
Human-β-actin	CACCATTGGCAATGAGCGGTTC	AGGTCTTTGCGGATGTCCACGT

### Western blotting

The protein levels were quantified using Western blotting. Proteins extracted from tissues or cells were separated on 10% or 12% SDS-PAGE and blotted onto the PVDF membrane (IPVH00010, Merck Millipore, Billerica, MA, USA) for 2 h at 250 mA. The membrane was blocked with 5% non-fat milk in TBS for 1 h at room temperature (RT), and then inoculated overnight with corresponding primary antibodies at 4°C. The blots were rinsed thrice in TBS enriched with 0.1% Tween 20 for 10 minutes and then inoculated with HRP-labelled secondary antibody (1:2000, AS014, Abclonal) for 1 hour in TBS enriched with 0.1% Tween 20 at RT. Immunoreactivity was detected through chemiluminescence using the ECL reagent. The primary antibodies used for Western blotting were: SIRT1 (1:2000, ab110304, Abcam), Beclin1 (1:2000, A7353, Abclonal), P62 (1:2000, A19700, Abclonal), Atg14 (1:2000, A7526, Abclonal), LC3 (1:2000, A19665, Abclonal), A19665, (1:50000, AC026, Abclonal).

### Luciferase reporter assay

Cells were co-transfected with luciferase vectors carrying wild-type or mutant 3′-UTR of Atg14 and miR-215 mimics or miR-Control using the Lipofectamine 3000 (2067450, Invitrogen, Carlsbad, CA, USA). Luciferase enzyme activity was determined using a Dual-Luciferase enzyme Reporter Assay System (RG027, Beyotime, Shanghai, China) at 48 hours after transfection.

### Statistical analyses

All statistical analyses were implemented in GraphPad Prism 8.0.1 software (GraphPad Software Inc., San Diego, CA, USA). Data are presented as Mean ± SD. Pairwise comparison between groups with normal distribution, homogeneous or different variance were performed using One-way ANOVA or LSD method. Groups with abnormal distribution, homogeneous or different variance were compared using the Wilcoxon rank sum tests or Kruskal Wallis method. *p* values less than 0.05 were considered to be statistically significant. ^#^*P* < 0.05, ^##^*P* < 0.01 and ^###^*P* < 0.001; ^*^P < 0.05, ^**^P < 0.01 and ^***^P < 0.001. ^#^ means significantly different from the control group; ^*^ means significantly different from the model group.

### Data availability statement

All data associated with this study are available from the first author on reasonable request.

## RESULTS

### EA and RSV ameliorated the AOM/DSS-induced CRC in mice

**Figure 1:**
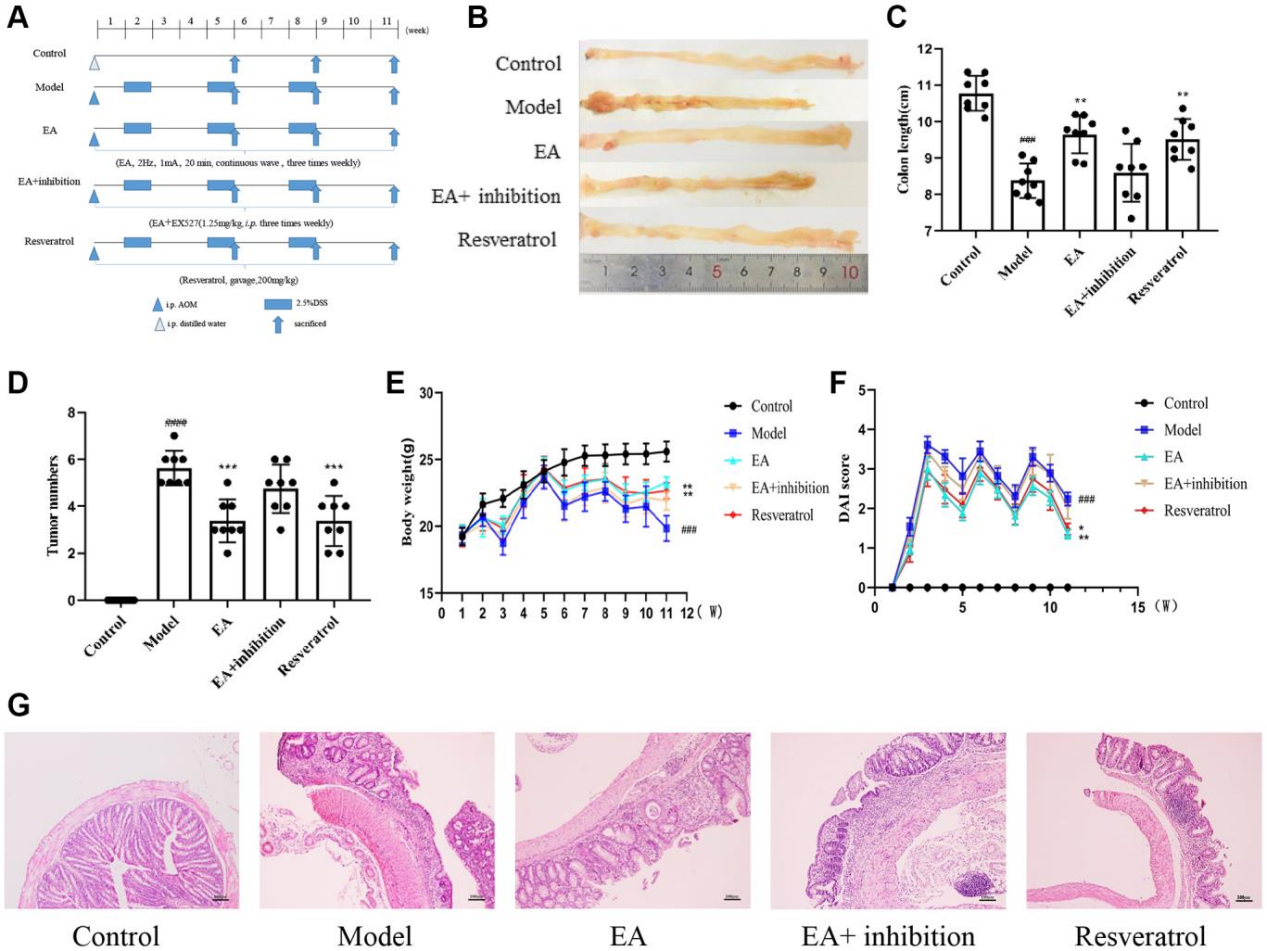
**EA and RSV ameliorated the AOM/DSS-induced CRC in mice.** (**A**) Experimental procedures. (**B**) Representative macroscopic images of colon tumor and colon measurements. (**C**) Colon length in each group. (**D**) Number of tumors in each group. (**E**) Changes in bodyweight and (**F**) DAI score. The notation "(W)" stands for "weeks”. (**G**) Representative HE staining of colorectal tissues (100× magnification). Data are expressed as mean ± SD (*n* = 8). ^###^*p* < 0.001, compared to the control group; ^*^p < 0.05, ^**^p < 0.01, and ^***^p < 0.001, compared to the model group.

It has been shown that EA can activate SIRT1 and reduce inflammation [32, 33]. In addition, the occurrence of CRC has been shown to be highly associated with SIRT1 [34] and inflammation [35]. To determine the protective mechanism of EA in CRC, a mouse model of colitis-induced CRC was established by feeding mice with azoxymethane (AOM)/dextran sodium sulfate (DSS) [36]. Mice were intraperitoneally injected with PBS or AOM and then given 2.5% DSS dissolved in drinking water. After an initial week, mice were given DSS-free water for two weeks, constituting one cycle. This three-cycle treatment regimen is outlined in the schematic diagram (Figure 1A). All other interventions and treatments were consistent across the groups. At the end of the experiment, intestinal tumors were observed with naked eye, confirming the success of CRC modeling (Figure 1B). Colon length (Figure 1C) and number of tumors (Figure 1D) were measured after dissection in the five groups at week 11 because colon shortening is often considered a visual index that reflects the severity of colorectal inflammation. As expected, treatment with AOM/DSS significantly increased the number of tumors (*p* < 0.001) and reduced the colon length (*p* < 0.0001). These parameters were not altered in the EA + inhibition group. Mice treated with EA and resveratrol showed significantly reduced number of tumors (*p* < 0.001) and increased colon length (*p* < 0.01). The body weight of mice increased gradually in the normal group over the observation period while the body weight and DAI of mice in other groups increased periodically. The DSS intervention led to a rapid decrease in mouse body weight, which then started to slowly increase after one week of intervention. Over the course of the three DSS cycles, there was a significant increase in body weight (Figure 1E). The DAI score was notably reduced (Figure 1F) following both resveratrol and EA treatments (*p* < 0.01), as compared to the model group. However, the difference in *DAI* score between the EA + inhibition group and model group were not significant (*p* > 0.05). These results illustrated that the effects of EA were similar to those of resveratrol of SIRT1, which could increase the body weight of mice and decrease the score of DAI under the intervention of DSS.

In the control group, HE staining revealed intact colorectal intestinal mucosa. However, in the Model and EA + inhibition groups, the intestinal mucosal epithelium appeared less intact. The glandular structure was disordered or even absent, and there was noticeable infiltration of inflammatory cells. Additionally, the crypts disappeared, cells displayed uneven sizes, and the nuclei appeared large and heavily stained. In the EA and resveratrol group, the intestinal mucosa was relatively intact, some epithelial cells were detached, the glandular arrangement was still regular, accompanied by mild to moderate inflammatory cell infiltration and submucosal edema, and epithelial cell morphology was still observed (Figure 1G).

### EA attenuates intestinal inflammation in CRC mice

**Figure 2:**
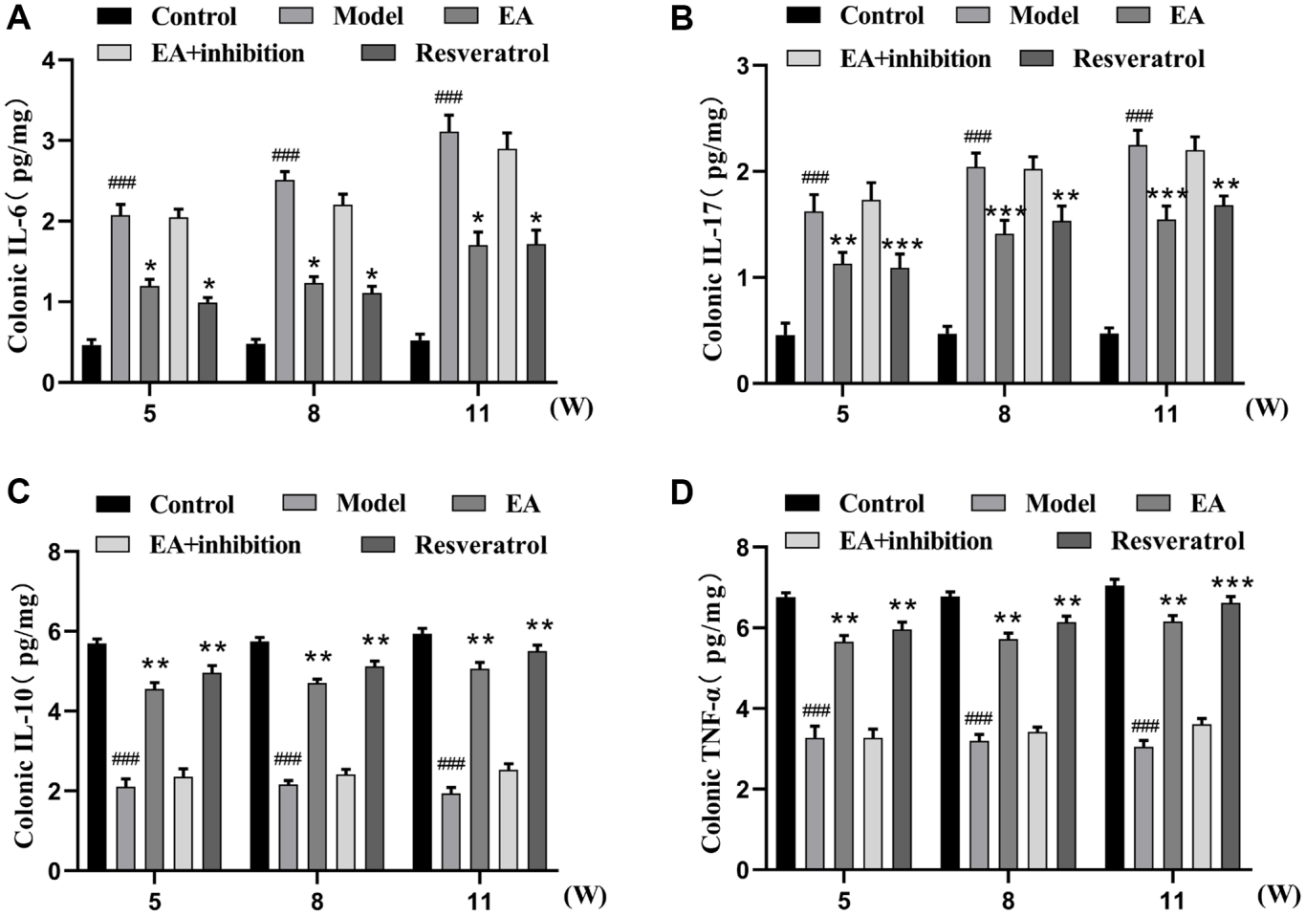
**EA decoction decreases the level of inflammation in colorectal tissue.** (**A**) IL-6 expression level. (**B**) IL-17 expression level. (**C**) IL-10 expression level. (**D**) TNF-α expression level. Three mice were analyzed in each group. Data are presented as mean ± SD from three independent experiments. ^#^*p* < 0.05, ^##^*p* < 0.01, ^###^*p* < 0.001, compared to the control group; ^*^p < 0.05, ^**^P < 0.01, and ^***^p < 0.001, compared to the model group.

Chronic inflammation has been shown to promote of colitis-induced colon carcinogenesis. IL-6 and IL-17 are proinflammatory cytokines whereas IL-10 and TNF-α are anti-inflammatory cytokines that regulate the development of CRC. Therefore, we investigated that EA inhibited CRC by augmenting the anti-inflammation. Changes in cytokine levels in colonic tissues were measured with ELISA kits. As is shown in Figure 2, inflammation level was lower in the control group compared to the model group (*p* < 0.001). The expression of IL-6 and IL-17 (Figure 2A, 2B) in the EA group and resveratrol group was significantly lower compared to that in the model group, while the expression of IL-10 and TNF-α (Figure 2C, 2D) in the EA group and resveratrol group was significantly higher compared with that in the model group. The levels inflammation in EA + inhibition group was slightly lower related to that of the model group, but the difference was not significant (*p* > 0.05).

### EA promotes SIRT1 expression and autophagy in CRC mice

**Figure 3:**
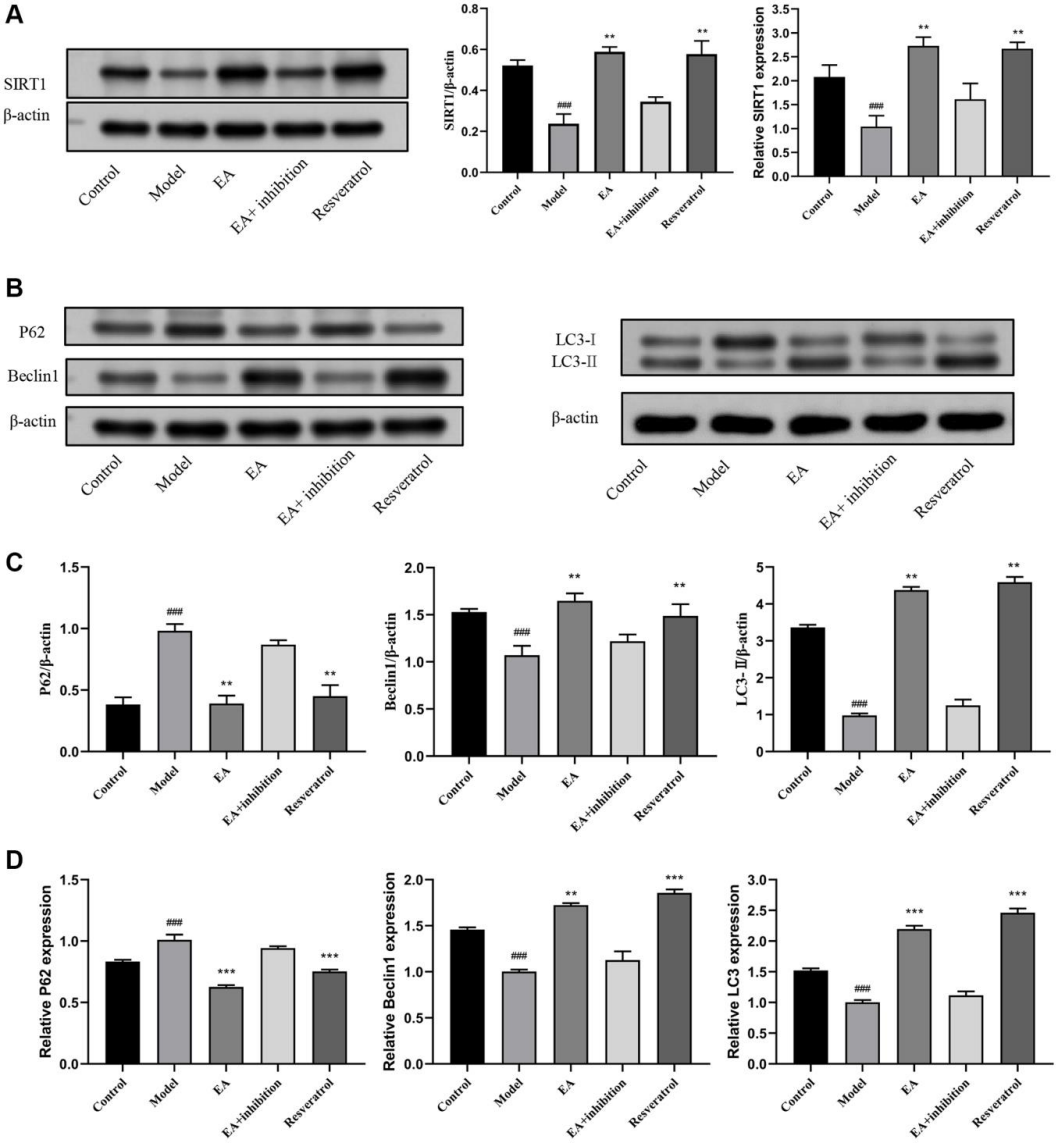
**EA promotes SIRT1 expression and autophagy.** (**A**) Western blotting and RT-qPCR and evaluation of SIRT1 expression in colon tissues. (**B**) Representative Western blotting graphs showing the expression of P62, Beclin1, and LC3 in colon tissues. (**C**) Quantitation of the expression contents of P62, Beclin1, and LC3. (**D**) RT-qPCR evaluation of the expression of P62, Beclin1 and LC3. Data are presented as mean ± SD (*n* = 8). ^#^*p* < 0.05, ^##^*p* < 0.01, ^###^*p* < 0.001, compared to the control group; ^*^p < 0.05, ^**^p < 0.01, and ^***^p < 0.001, compared to the model group.

In further experiments, we explored whether the benefits of EA on CRC were mediated by increased SIRT1 overexpression and autophagy activation. The expression of SIRT1 protein was significantly lower in colorectal tissue of the model group compared with that of the control group (*p* < 0.01). Treatment with EA increased the protein expression of SIRT1 significantly (*p* < 0.01), demonstrating that EA upregulates SIRT1 expression. Results showed that SIRT1 expression in the resveratrol group was higher than in the control group, indicating that resveratrol effectively increased the protein expression of SIRT1 in colorectal tissue. The expression of SIRT1 was not difference between the model group, EA + inhibition group, and EA group (*p* > 0.05), suggesting that the inhibitor partially suppressed the effect of EA on the expression of SIRT1 protein and EA activated SIRT1 (Figure 3A). The content of Beclin1 and LC3 in the intestinal tissue of the model group was significantly lower than in the control group (*p* < 0.001), while the expression of P62 was higher (*p* < 0.01), indicating that autophagy was inhibited in the model group. After treatment with EA, the expression of Beclin1 and LC3 increased significantly, whereas that of P62 decreased (*p* < 0.01), indicating that EA activated autophagy. In the resveratrol group, the expression of autophagy-linked proteins was significantly increased, indicating that SIRT1 overexpression promoted the expression of autophagy-linked proteins in colorectal tissue. In contrast, the expression of Beclin1 and LC3 was increased whereas that of P62 was downregulated in the EA + inhibition group (Figure 3B–3D).

After confirming the role of EA in regulating autophagy at the protein level, we further verified it at the tissue level. As shown in Supplementary Figure 1, after EA treatment, the expression of Beclin1 (*p* < 0.001) and LC3 (*p* < 0.001) was increased, and the expression of P62 (*p* < 0.01) was decreased. Application of the SIRT1 agonist resveratrol synergized the effect of EA to enhance the occurrence of autophagy (Supplementary Figure 1).

### SIRT1 represses miR-215 expression

**Figure 4:**
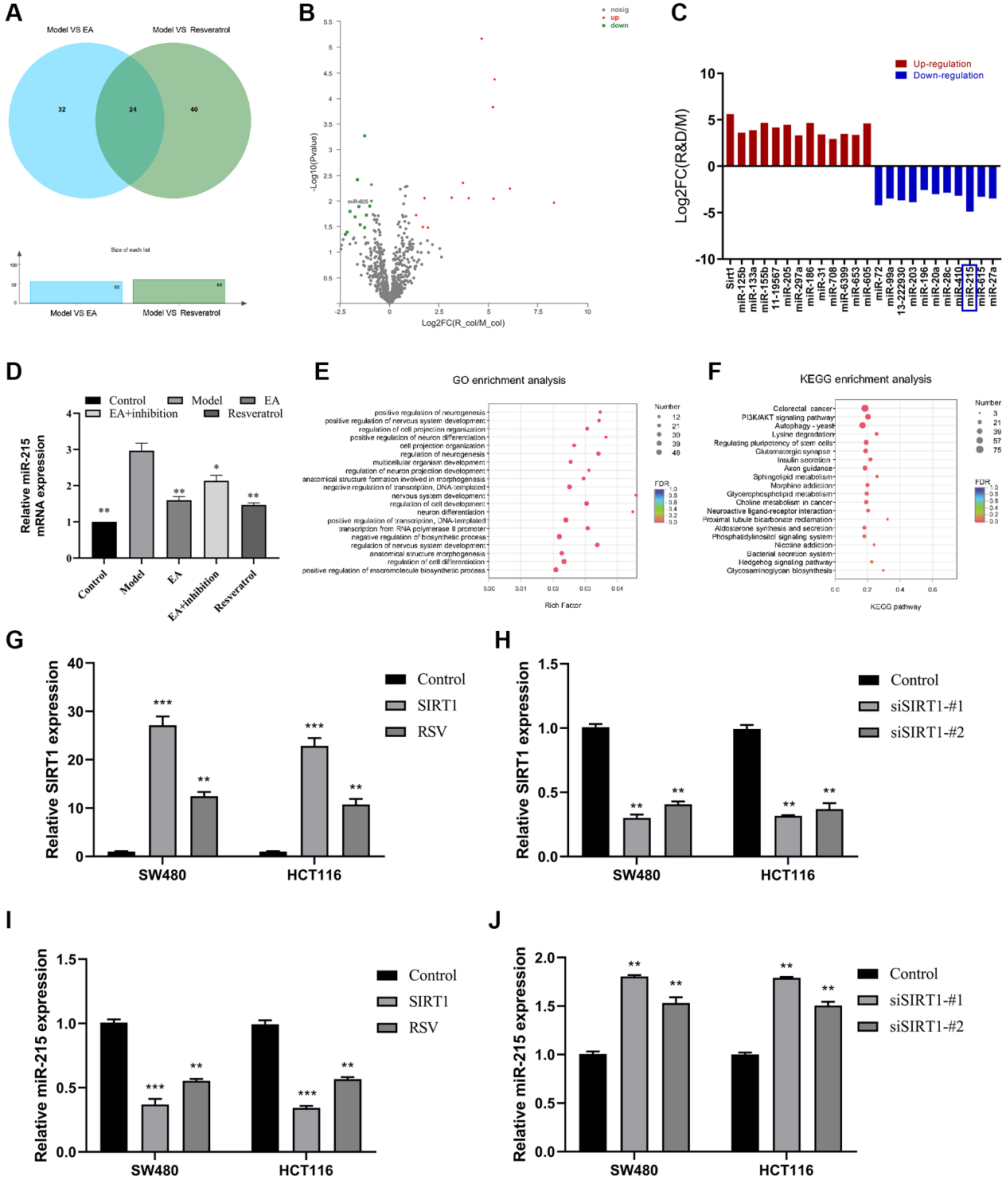
**SIRT1 represses miR-215 expression.** (**A**) Venn diagram showing miRNAs co-regulated by Model vs. EA and Model vs. resveratrol; (**B**) Volcano plots showing differentially expressed miRNAs (Red: high expression; green: low expression). (**C**) Differential gene expression. (**D**) Relative expression level of miR-215 in the mice colorectal tissue by RT-qPCR detection. (**E**) GO enrichment assessment. (**F**) KEGG enrichment assessment. (**G**) mRNA expression of SIRT1 after overexpression of SIRT1 in SW480 and HCT116. (**H**) mRNA expression of SIRT1 after inhibition of SIRT1 in SW480 and HCT116. (**I**) mRNA expression of miR-215 after overexpression of SIRT1 in SW480 and HCT116. (**J**) mRNA expression of miR-215 after inhibition of SIRT1 in SW480 and HCT116. Three mice were analyzed in each group. Data indicate mean ± SD from three independent experiments. ^*^p < 0.05, ^**^p < 0.01, and ^***^p < 0.001, compared to the control group.

Several microRNAs have been shown to be important regulate CRC. Moreover, EA can treat a variety of diseases by targeting miRNA. In this study, we explored the targets of EA in the intestinal tissue from the CRC model. The result showed 24 miRNAs with obvious differences and co-expression were obtained (Figure 4A, 4B). The changes in expression of the 24 candidate genes after SIRT1 overexpression was further detected by qPCR (Figure 4C), and miR-215 expression level was dramatically down-regulated (more than 2 times) (Figure 4D). Further enrichment analysis of GO and KEGG also showed that it was associated with autophagy (Figure 4D–4F). Subsequently, SIRT1 overexpression plasmid (SIRT1) and SIRT1-silencing vectors (siSIRT1) were constructed and transiently transfected into HCT116/SW480 cells. The RT-qPCR analysis demonstrated that transfection with the SIRT1 overexpression plasmid led to an increase in SIRT1 expression (Figure 4G). Conversely, siSIRT1 effectively suppressed the rise in SIRT1 levels (Figure 4H). Additionally, the expression level of miR-215 exhibited an inverse relationship with SIRT1 levels (Figure 4I, 4J).

### Atg14 is a miR-215 direct target

**Figure 5:**
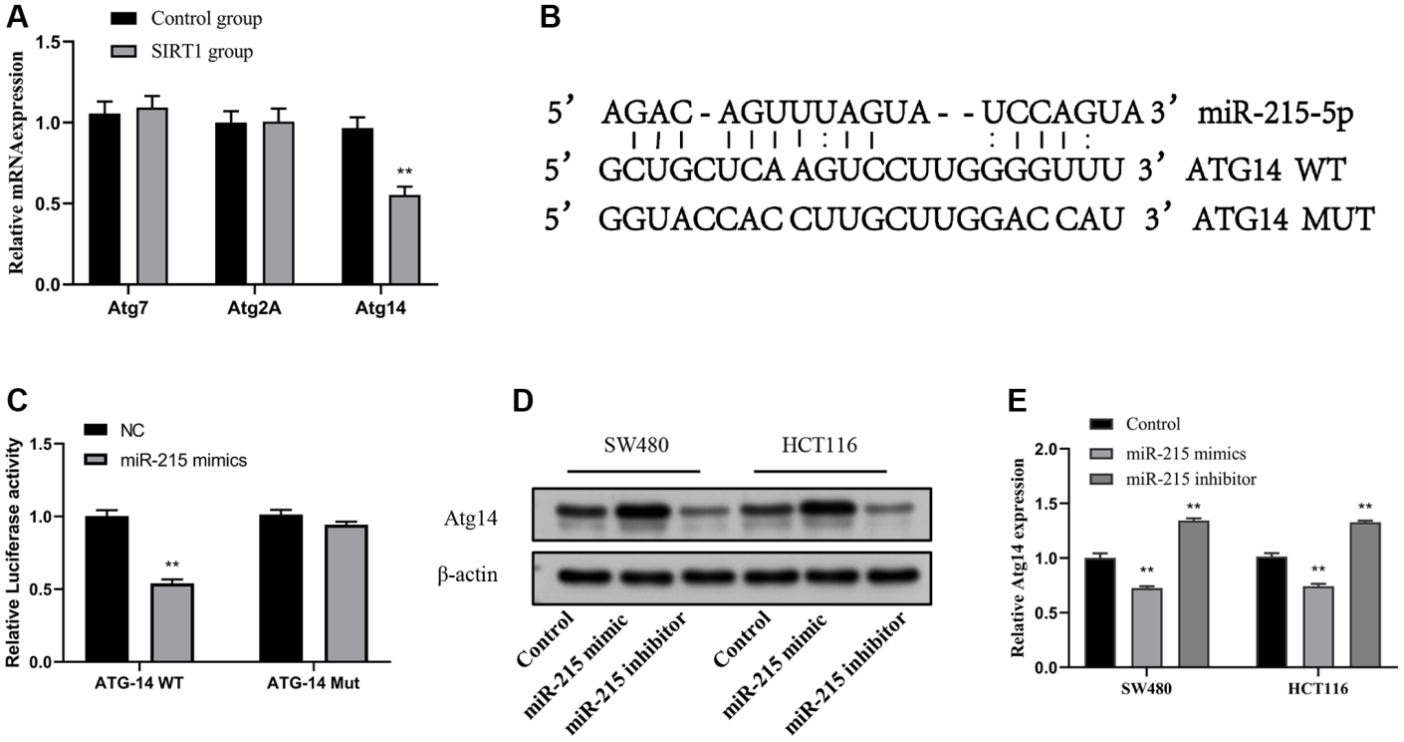
**Atg14 is a direct target of miR-215.** (**A**) RT-qPCR analysis of Atg gene expression after SIRT1 overexpression. (**B**) Schematic illustration of the predicted miR-215-5p docking sequences for Atg14. (**C**) The luciferase reporter activity of Luc-ATG14-WT/MUT was detected by dual luciferase reporter assay. (**D**, **E**) Transfection of mimic-miR-215-5p or inhibitor-miR-215-5p in SW480 and HCT116 cells to evaluate Atg14 proteins (**D**) and mRNA (**E**) levels. Data are presented as mean ± SD from three independent experiments. ^*^p < 0.05, ^**^p < 0.01, and ^***^p < 0.001, compared to the control group.

To elucidate the mechanism of miR-215 promoting autophagy, we found that there are three kinds of ATGs (Atg7, Atg2A and Atg14) that may bind to it, through the online prediction software TargetScan and RNAhybrid (Figure 5A). The results of RT-qPCR experiment indicated that Atg14 showed a high expression trend after resveratrol and overexpression of SIRT1 plasmid. To investigate the binding between miR-215 and Atg14, we utilized a luciferase reporter plasmid with both wild-type and mutated docking sites (Figure 5B). To further evaluate whether Atg14 is the direct target of miR-215, pmiR-RB-ReportTM-WT-Atg14 and pmiR-RB-ReportTM-MUT- Atg14 were co-transfected with miR-215 mimic or inhibitor into SW480 and HCT116 cells. After treatment with miR-215 mimic, the relative luciferase activity in the reporter with wild type 3′UTR of Atg14 but not in the reporter harboring a mutated miR-215 binding site in the 3′UTR of Atg14 was significantly attenuated (Figure 5C). Western blotting and RT-qPCR experiments revealed that the expression of Atg14 protein (Figure 5D) and mRNA (Figure 5E) was significantly decreased in CRC cells following miR-215 mimic transfection, but it was up-regulated after miR-215 inhibitor transfection (Figure 5D, 5E). Meanwhile, we also examined the effects of EA on miR-215 and Atg14 in mouse colon tissue and found that EA and resveratrol inhibited miR-215 expression and promoted Atg14 expression, whereas EA + inhibitor showed no significant changes (Supplementary Figure 2).

### SIRT1 regulates autophagy through miR-215/Atg14 axis

**Figure 6:**
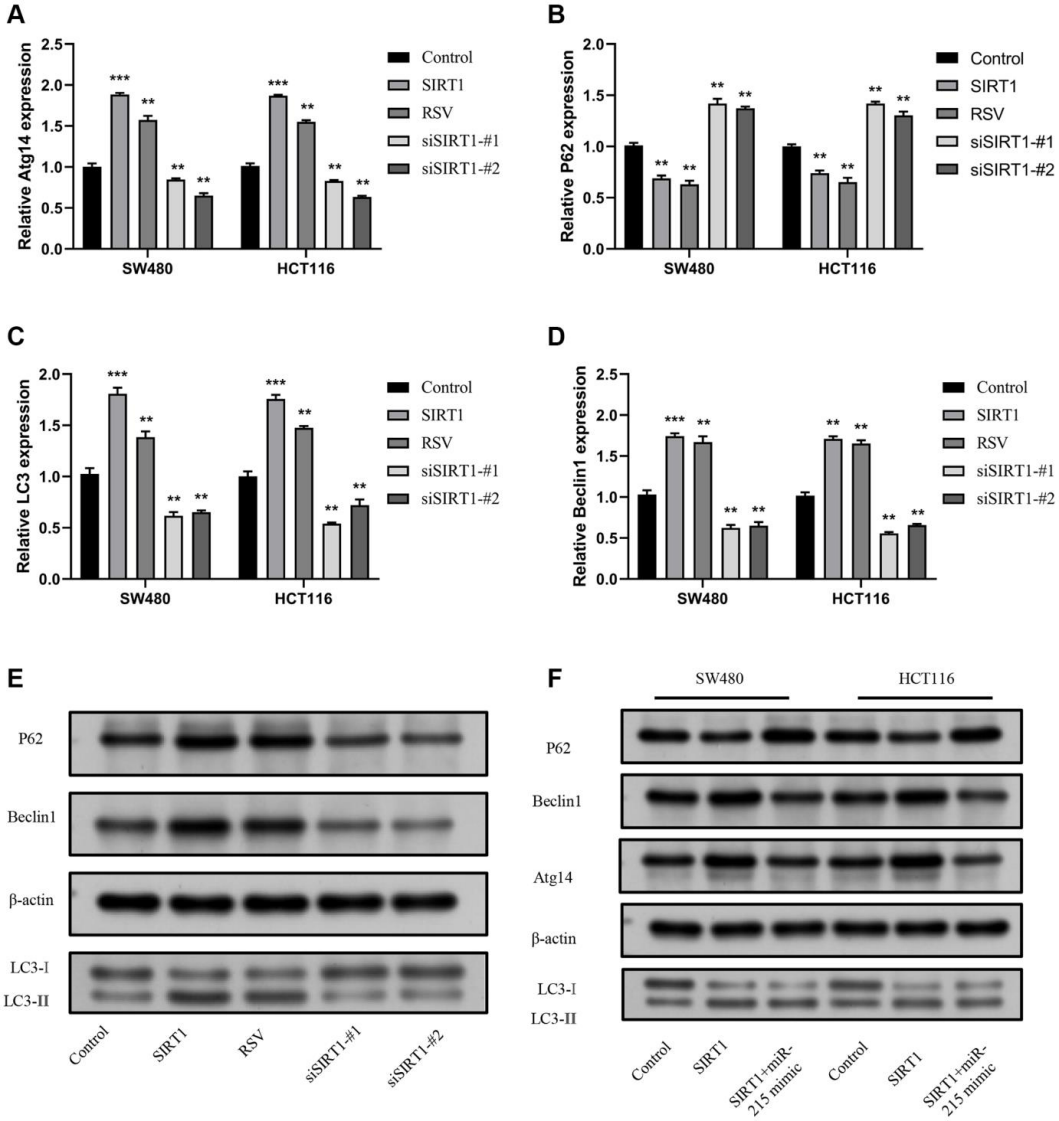
**SIRT1 promotes autophagy via the miR-215/Atg14 axis.** (**A**) Detection of the expression level of Atg14 mRNA following overexpression of SW480 and HCT116 and inhibition of SIRT1. (**B**–**D**) Detection of the expression level of autophagy-related molecules mRNA following overexpression of SW480 and HCT116 and inhibition of SIRT1. (**E**) Detection of the expression level of autophagy-related molecular proteins following overexpression and inhibition of SIRT1 in SW480 and HCT116. (**F**) Detection of the expression of Atg14 and other autophagy-related molecules following overexpression of SIRT1, and insertion of miR-215 mimics in SW480 and HCT116. Three mice were analyzed in each group. Data are presented as mean ± SD from three independent experiments. ^*^p < 0.05, ^**^p < 0.01, and ^***^p < 0.001, compared to the control group.

To explore the effect of SIRT1/miR-215/Atg14 axis on autophagy in CRC, the mRNA expression level of Atg14 was measured by RT-qPCR in SW480 and HCT116 cells after overexpression or knockdown of SIRT1. Results indicated that overexpression of SIRT1 increased Atg14 expression, while knockdown of SIRT1 decreased Atg14 expression (Figure 6A). Furthermore, the expression of autophagy- related molecule P62, Beclin1 and LC3 was detected. The findings indicated that overexpression of SIRT1 led to increased levels of Beclin1 and LC3, while P62 expression decreased. Conversely, when SIRT1 was knocked down, a statistically significant difference was observed (Figure 6B–6D). The same trends were observed by Western blot analysis (Figure 6E). Furthermore, we also performed the rescue experiment *in vitro*. In SW480, as well as HCT116 cells, the autophagy level of SIRT1 + miR-215 mimic group was lower in contrast with that of SIRT1 overexpression group, but higher in contrast with that of control group (Figure 6F). Overexpression of miR-215 expression remarkably inhibited the autophagy-induced effects on SIRT1. In summary, these results illustrate that the SIRT1/miR-215/Atg14 axis plays an important role in occurrence of autophagy in CRC.

## DISCUSSION

Acupuncture is an important component of the traditional Chinese medicine (TCM), with a history of more than three thousand years [37]. In recent decades, studies have shown that acupuncture can treat and improve symptoms of various diseases. According to the theory of meridians and collaterals, the specific combination of acupoints can alleviate inflammatory symptoms. This is why acupuncture is extensively used in the treatment of cancer-related pain [38], fatigue [39], and various other discomforting symptoms [40]. In our previous study, we found that EA inhibited inflammation via the NF-κB signaling pathways [41], and by activating SIRT1 [42, 43]. In the present study, our results indicated that EA delayed the growth of tumors, reduced the number of tumors, decreased DAI score, and suppressed inflammation level. It also promoted autophagy after activating SIRT1. Previously, SIRT1 was reported to participate in the deacetylation of histones, transcription factors, as well as signaling proteins involved in the regulation of metabolic and stress-response pathways [44, 45]. The role of SIRT1 in cancer biology has been demonstrated. Research has shown that SIRT1 regulates stress responses indicating that it can also be a tumor suppressor [46]. Here, we uncovered that SIRT1 inhibited CRC both *in vitro* and *in vivo*. Moreover, activation of SIRT1 delayed tumor growth and reduced tumor number *in vitro*. Elsewhere, SIRT1 expression was reported to inhibit the migration and invasion of CRC cells [47], and was linked to better overall survival of CRC patients [48]. This may explain the decrease of SIRT1 expression was decreased in CRC mice.

Furthermore, AOM/DSS treatment inhibited autophagy by increasing LC3-II and P62 expression and suppressing Beclin1 expression. LC3-II indicates the number of autophagosomes, and P62 is an autophagy cargo protein. The expression of Beclin1 was downregulated in CRC whereas Beclin1 overexpression and activation of autophagy inhibits tumor growth [49]. EA treatment led to an increase in the number of autophagosomes and the degradation of autophagy cargo proteins, providing confirmation that EA treatment enhanced autophagy [50]. Autophagy is categorized into three types: macroautophagy, microautophagy, and molecular chaperone-triggered autophagy. Among these, macroautophagy is considered the predominant type of autophagy, and it can either promote or inhibit tumor development [51]. Currently, the role of autophagy in the progression of malignant tumors is poorly understood. Our results indicate that EA inhibits CRC by activating SIRT1 and promoting autophagy. Notably, autophagy was not affected in EA + inhibition group. The RSV group exhibited a similar effect to the EA group. Therefore, we postulated that EA might induce apoptosis in CRC cells by suppressing autophagy. Nevertheless, the precise mechanism through which EA modulates autophagy remained unclear.

The miRNA, a subset of small non-coding RNA, can bind to the 3′UTR of mRNA to alter diverse effects on cellular functions. Research has shown that miRNA regulates several biological processes and these have been implicated in multiple diseases, including cancer. They are specifically modulate the basic hallmarks of cancer, including cell proliferation, angiogenesis [52], EMT [53], metabolism [54] and autophagy [55]. The expression patterns of miRNAs are potential biomarkers of various diseases [56]. Animal studies have documented that miRNAs may mediate the effects acupuncture [57]. The therapeutic impact of EA is extensive and encompasses various aspects. Examining the miRNA regulatory mechanism in association with EA can provide deeper insights into its underlying workings. RNA sequencing analysis showed that both EA and resveratrol treatments led to the upregulation or downregulation of several miRNAs compared to the model group. Further RT-qPCR analysis confirmed that both EA and resveratrol suppressed the expression of miR-215, which exhibited a negative correlation with SIRT1 expression. This further demonstrated that EA inhibited the expression of miR-215 by activating SIRT1. miR-215 is a target of p53 that is up-regulated [58] following DNA damage. It inhibits tumor development by regulating tumor microenvironment remodeling [59] and tumor stem cell differentiation [60]. However, some studies have shown that miR-215 enhances carcinogenesis by inhibiting tumor suppressor genes [61, 62]. The high expression of mir-215 in CRC has been shown to be associated with poor overall survival rate. Other experimental results have demonstrated that miR-215 may function as an oncogene in CRC by inhibiting dicer expression [63].

Moreover, we identified a key functional cascade SIRT1/miR-215/Atg14 that regulates autophagy in CRC. *Atg14* is a core modulatory protein that participates in the initiation of autophagosomal particles [64]. In this study, dual luciferase reporter gene assay showed that Atg14 was a target of miR-215. Other studies have demonstrated that Atg*14* can activate autophagy and overcome insulin resistance in human hepatoma carcinoma cells [65]. Importantly, our *in vivo* data showed that Atg14 expression was upregulated and autophagy was activated due to EA-induced SIRT1 activation. These changes resulted in the inhibition of tumor growth. In addition, *in vitro* experiments uncovered that miR-215 suppressed autophagy of CRC cells by upregulating SIRT1 expression.

This study has the following limitations. In our initial investigation, we found that both the sham acupuncture group and the non-acupoint control group did not exhibit a significant impact on inflammatory obesity when compared to the acupuncture group. However, to further establish the efficacy of Zusanli (ST 36) and Fenglong (ST 40) acupoints in CRC, it is crucial to include sham acupuncture and non-meridian acupoints in future experiments. This additional step will enhance the comprehensiveness of our study. Secondly, the effects of EA on CRC are mediated by multiple systems, targets and pathways. However, other molecules and pathways may be involved. In this study, we only explored the intervention mechanism based on the autophagy pathway, other pathways need to be further investigated. Nonetheless, our findings provide novel insights into the therapeutic mechanisms underlying the beneficial effects of EA on CRC. The observed effects were supported by both cell function experiments and animal experiments. Specifically, we demonstrate that EA meliorates inflammation and promotes autophagy in colorectal cancer via SIRT1/miR-215/Atg14 Axis. This discovery helps us better understand the anticancer effect of EA and its ability to regulate SIRT1, leading to regulation of autophagy and delay of carcinogenesis.

## CONCLUSION

**Figure 7:**
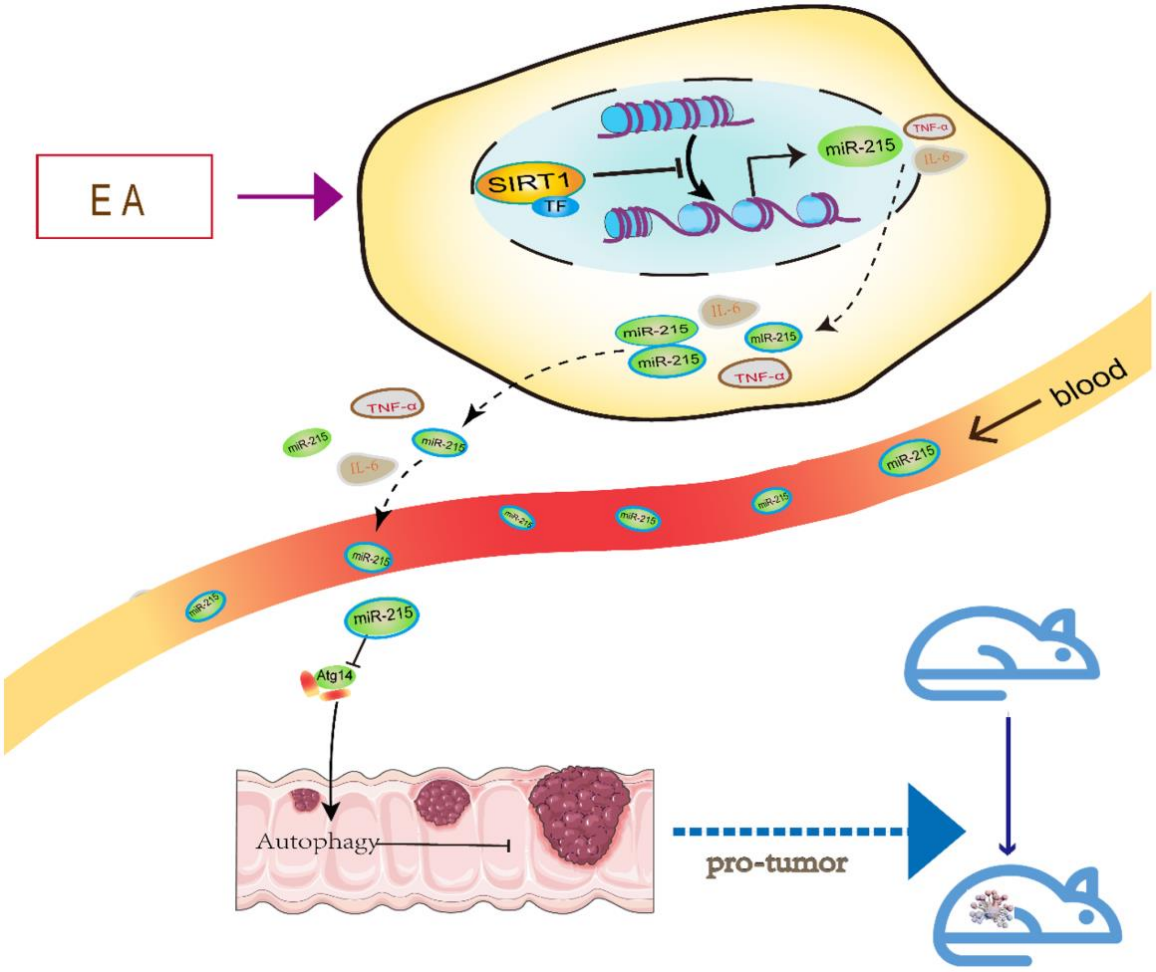
A schematic model illustrating the protective effect of EA on CRC via the SIRT1/miR-215/Atg14 axis.

The data shown in the graphical abstract (Figure 7) demonstrates that EA activates SIRT1 to inhibit miR-215 and restore Atg14 expression. Through this mechanism, SIRT1 promotes autophagy and inhibits CRC. These results demonstrate that EA activates autophagy of CRC cells via the SIRT1/miR-215/Atg14 axis. Thus, it is a potential target for the treatment of CRC.

## Supplementary Materials

Supplementary Figures
