# Supplementary material for: Electroacupuncture ameliorates AOM/DSS-induced mice colorectal cancer by inhibiting inflammation and promoting autophagy via the SIRT1/miR-215/Atg14 axis
Source: Aging (Albany NY). 2023 Nov 22;15(22):13194–212. doi: 10.18632/aging.205236 (PMC10713401; doi:10.18632/aging.205236)
Supplement: Supplementary Figures [file aging-15-205236-s001.pdf]

SUPPLEMENTARY FIGURES

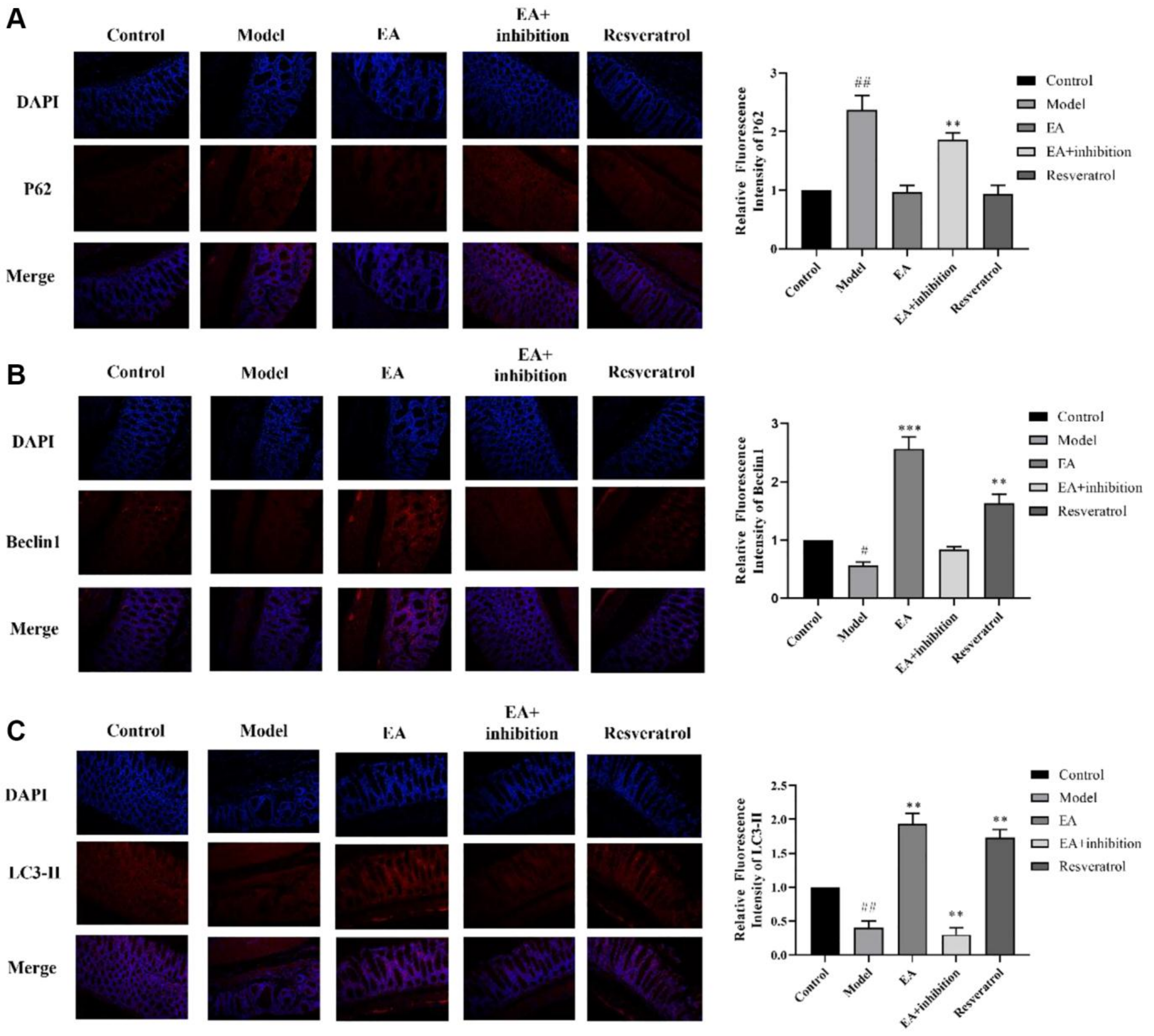

Supplementary Figure 1. EA promotes autophagy in colon tissue. (**A**–**C**) Immunofluorescence of P62, Beclin 1, LC3-II expression in colon tissue and analysis of the results of the immunofluorescence. $^{\#}p < 0.05$, $^{\#\#}p < 0.01$, compared to the control group; $^{*}p < 0.05$, $^{**}p < 0.01$, and $^{***}p < 0.001$, compared to the model group.

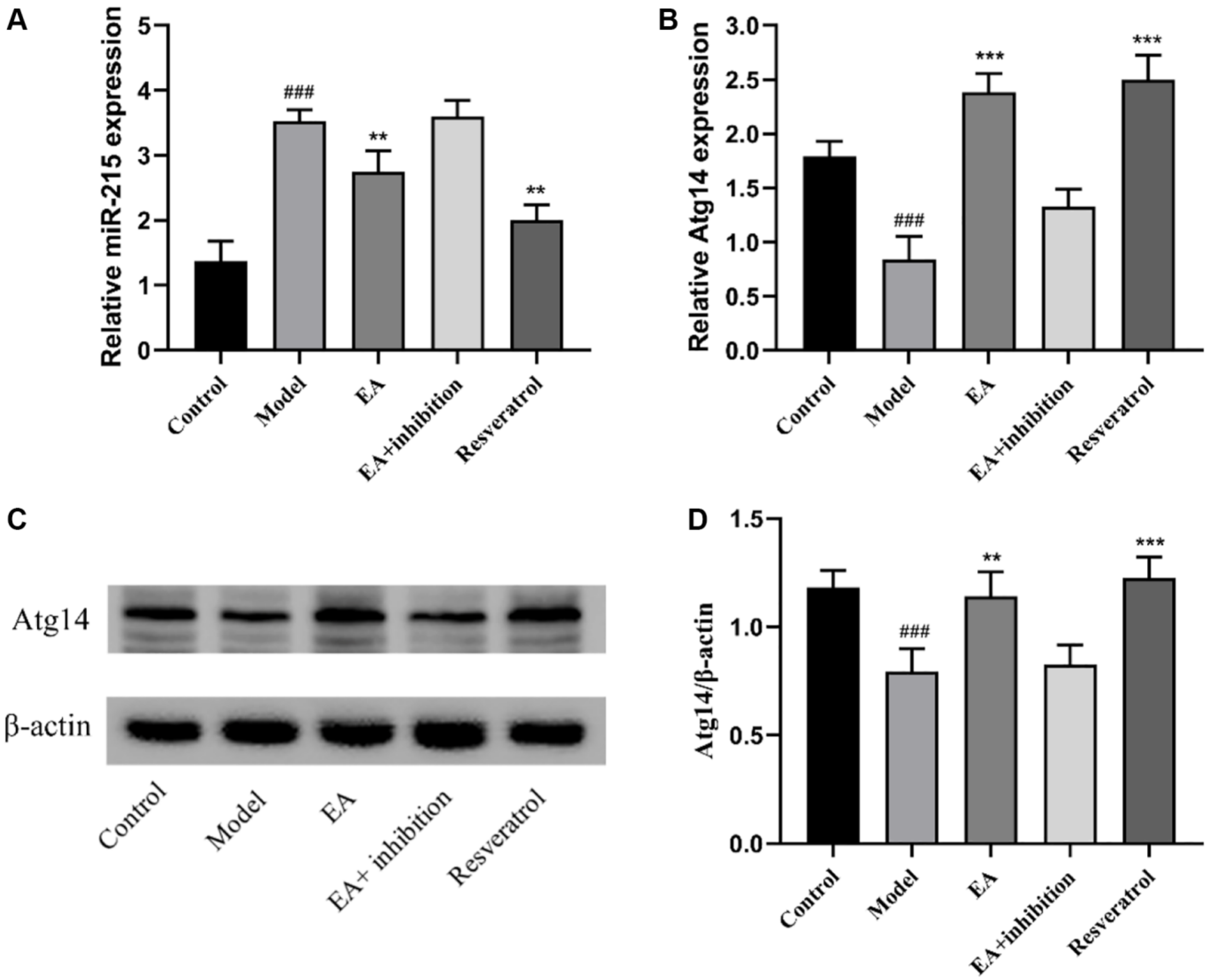

Supplementary Figure 2. EA inhibited miR-215 expression and promoted Atg14 expression. (**A**, **B**) Expression levels of miR-215 and Atg14 by RT-qPCR. (**C**) Representative Western blotting graphs showing the expression of Atg14 in colon tissues. (**D**) Quantitation of the expression contents of Atg14. Data are presented as mean ± SD (n = 3). $^{###}p < 0.001$, compared to the control group; $^{**}p < 0.01$, and $^{***}p < 0.001$, compared to the model group.
